# Early Diabetic Nephropathy and Retinopathy in Patients with Type 1 Diabetes Mellitus Attending Sudan Childhood Diabetes Centre

**DOI:** 10.1155/2020/7181383

**Published:** 2020-11-24

**Authors:** Hana Ahmed, Tayseer Elshaikh, Mohamed Abdullah

**Affiliations:** ^1^Department of Paediatric and Child Health, Faculty of Medicine, University of Khartoum, Khartoum, Sudan; ^2^Department of Ophthalmology, Jabir Abu Eliz Diabetes Centre, Khartoum, Sudan

## Abstract

**Objective:**

Data on microvascular complications in children and adolescents with type 1 diabetes mellitus (T1DM) in Sudan are scarce. This study was aimed at determining the prevalence of diabetic nephropathy (DN) and retinopathy (DR) and their relationship to certain risk factors in children with T1DM attending the Sudan Childhood Diabetes Centre. *Design and Methods*. A clinic-based cross-sectional study of 100 patients with T1DM aged 10-18 years. Patients with disease duration exceeding 5 years if the onset of diabetes was prepubertal and 2 years if it was postpubertal were included. Relevant sociodemographic, clinical, and biochemical information was obtained. Blood pressure was measured. The patients were screened for DN and DR using urinary microalbumin estimation and fundus photography, respectively.

**Results:**

The frequency of microalbuminuria and diabetic retinopathy was 36% and 33%, respectively. Eleven percent had both retinopathy and microalbuminuria. Seven percent of the patients were found to be hypertensive. Patients with diabetic retinopathy had significantly higher HbA1c levels (*p* = 0.009) and longer diabetes duration (*p* = 0.02) than patients without retinopathy. Logistic regression showed that high HbA1c (odds ratio (OR) 0.83, confidence interval (CI) 0.68-1.00, *p* = 0.04), but not age, duration, ethnic group, BMI, blood pressure, and presence of nephropathy, was an independent risk factor for retinopathy. Likewise, high blood pressure (OR 6.89, CI 1.17-40.52, *p* = 0.03), but not age, duration, ethnic group, BMI, HbA1c, and presence of retinopathy, was a predictor for nephropathy.

**Conclusion:**

High prevalence of incipient DN and early stages of DR were observed in this study. Longer diabetes duration and higher HbA1c were associated with the presence of diabetic retinopathy. High blood pressure was a risk factor for DN. So regular screening for these complications and optimization of glycemic control are needed.

## 1. Introduction

Type 1 diabetes mellitus (T1DM) can lead to microvascular complications including nephropathy, retinopathy, and neuropathy. These chronic complications lower the quality of life and cause premature death in patients with diabetes [1–3]. Clinically evident diabetes-related microvascular complications are rare in childhood and adolescence. However, early functional and structural abnormalities may be present a few years after the onset of the disease [2]. So screening for these complications is important to identify their occurrence at an early stage.

These microvascular complications of T1DM are associated with certain risk factors like age, ethnicity [4–6], age at onset, diabetes duration, height, BMI, and puberty [1, 2, 7–11].

Other modifiable risk factors include high blood pressure (BP) [2, 12, 13] and long-term glycemic control [8, 9, 14]. The latter was found by many studies to be the most important risk factor for the development of diabetic microangiopathies [8, 14]. Based on the results of the DCCT (Diabetes Control and Complications Trial) and its follow-up study EDIC (Epidemiology of Diabetes Interventions and Complications), it was recommended to optimize glycemic control as early and close to normal as possible in all patients with T1DM to prevent the development and progression of microvascular complications [3].

There is a paucity of studies on microvascular complications in children and adolescents with T1DM in sub-Saharan Africa. However, all the African published studies showed high prevalence rates in patients with relatively short duration and at a young age [15–17]. Whether this is due to the expected suboptimal glycemic control in developing economies or genetic elements remains a question for further studies. In Sudan, one unpublished study, from 2012, examined young patients with diabetes aged 11-19 years. It reported microalbuminuria in 25% and macroalbuminuria in 15% of the participants [18].

This study was aimed at determining the prevalence and risk factors for diabetic nephropathy and retinopathy among patients with T1DM attending the Sudan Childhood Diabetes Centre.

## 2. Research Design and Methods

This is a cross-sectional study of consecutively enrolled 100 children with T1DM aged 10-18 years attending the Sudan Childhood Diabetes Centre. The study lasted from February to September 2018. The Sudan Childhood Diabetes Centre is a specialized centre with a comprehensive multidisciplinary team that includes trained endocrinologists, dentists, ophthalmologists, diabetes nurses, diabetes educators, psychologists, dieticians, and social workers. The centre provides free insulin and glucometers either free or at a low cost. Ethical approval was obtained from the Sudan Childhood Diabetes Centre. Informed consent was obtained from the patient and/or his parents.

The diagnosis of T1DM was mostly made by the treating paediatric endocrinologist in the centre clinically, as routine testing for autoantibodies or c-peptide is expensive.

Patients aged ≥10 years with diabetes duration˃5 years if the onset was prepubertal and ˃2 years if it was postpubertal were included [2]. The assessment was performed during the clinic visit, using a validated structured questionnaire. Sociodemographic data include age, sex, residence, and the tribe of the patient using the most accepted ethnic classification of the Sudanese population [19]. Additionally, data on age at onset, duration of diabetes, state of home blood glucose monitoring, dose, and regimen of insulin were collected. A conventional insulin regimen was defined as twice per day premixed insulin plus prelunch regular insulin, while an intensive insulin regimen (MDI = multi-insulin injection regimen) as insulin glargine once/day or NPH twice a day as basal plus regular or rapid-acting insulin before each meal.

Anthropometric measurements, pubertal staging, and blood pressure measurement were done during the interview.

### 2.1. Blood Pressure Measurement

To assess blood pressure (BP), the patient sat down for a few minutes for rest before two measurements with a 5-10-minute interval, using an aneroid sphygmomanometer, were done. The Korotkoff phases I and V were the systolic and diastolic readings, respectively. Blood pressure was categorized according to BP tables from the fourth report of the national high blood pressure education program working group on the diagnosis, evaluation, and treatment of high blood pressure in children and adolescents, using age and height percentiles, with normotension defined as blood pressure under the 90th percentile, prehypertension systolic blood pressure (SBP) and/or diastolic blood pressure (DBP) 90th to 95th percentile, and hypertension greater than the 95th percentile [20].

### 2.2. Anthropometric Measurements

Weight was measured using weighing scales to the nearest 0.1 kg. Height was measured using a stadiometer and was expressed in centimeters with no decimal. BMI was categorized according to the Centers for Disease Control and Prevention (CDC) age- and sex-specific growth charts. The following categories were used: underweight: <5th percentile, normal weight: 5th to 85th percentile, overweight: 85th to 95th percentile, and obese: >95th percentile [21]. The sexual maturity rating of the children was determined using the Tanner scale [22].

### 2.3. Laboratory Evaluation

The most recent HbA1c was obtained from the patients' record. HbA1c was performed in the centre's laboratory with a point-of-care ichromἀ analyzer (company). Then, the patients were classified according to ISPAD 2018 (International Society for Pediatric and Adolescent Diabetes) guidelines which consider HbA1c < 7.5% to be the level for optimal control in resource-limited environments [23].

Urine was examined for the presence of microalbumin in a spot random urine sample, using quantitative immunoturbidimetry of the HemoCue brand (company). Any reading less than 20 mg/l, between 20 mg/l and 200 mg/l, and more than 200 mg/l was considered negative, microalbuminuria, and macroalbuminuria, respectively. Testing on any febrile child, a child with a urinary tract infection, or menstruating female patients was postponed till the next visit. As per ISPAD Clinical Practice Consensus Guidelines 2018 [24], a patient was considered to have persistent microalbuminuria if he had two out of three positive results in the 3-6-month period.

### 2.4. Retinopathy Diagnosis

Retinopathy was assessed by nonmydriatic two-field digital fundus photography using a fundus camera of the DRS brand (Digital Retinography System) manufactured by CenterVue. The photography was carried out by an experienced optician, and the comments on the photos were made by an ophthalmologist experienced in diabetic retinopathy. Both the optician and the ophthalmologist were masked to the clinical status of the patient.

The classification of diabetic retinopathy was based on the Early Treatment Diabetic Retinopathy Study (ETDRS) classification [25], which regards any diabetes-related retinal lesion, commonly microaneurysms, as the onset of diabetic retinopathy. This is following the latest ISPAD recommendation for retinopathy screening [24].

### 2.5. Statistical Analysis

Descriptive statistics were reported as mean ± standard deviation when normally distributed and as median (interquartile range) for nonparametric data. Clinical characteristics and complication rates were compared using chi-square tests or Fisher's exact test for categorical variables, *t-*tests for normally distributed continuous data, and Mann-Whitney *U* tests for skewed data. Comparison between more than two groups was performed using the Kruskal-Wallis test. The distribution of continuous data was tested for normality by the Kolmogorov-Smirnov test. Logistic regression was performed with the presence of DR and DN as dependent variables. The independent variables were age, diabetes duration, ethnic group, BMI, blood pressure, HbA1c, and the presence of the other complication (DR for DN and vice versa). Because of the significant collinearity between duration and age at onset and age and Tanner stage, age at onset and Tanner stage were omitted from the final model. Results are reported as odds ratio (OR) and 95% CI. All statistical tests were two-tailed with a *p* value < 0.05 considered significant. Data were analyzed using the Statistical Package for the Social Sciences program (IBM-SPSS) version 23.

## 3. Results

### 3.1. Sociodemographic and Clinical Characteristics of the Participants

A total of one hundred patients with T1DM constituted the study population; their sociodemographic, ethnic, and clinical characteristics are shown in Table 1. As shown, the majority were Afro-Asiatic (79%). Regarding the growth and pubertal status of the study population, the majority had normal BMI (71%) and were pubertal (94%). Only 2% were obese. Seven percent had hypertension, and 8% had prehypertension. The youngest patient with hypertension was 12 years old. Fifty-seven percent of the patients with hypertension and 50% of those with prehypertension had microalbuminuria.

Most patients (95%) had a glucometer for home blood glucose monitoring, yet only 10% were checking daily and 39% check only when symptomatic. The rest do 2-3 readings per day in fixed 2 days per week.

Regarding the glycemic control of the study population, the median HbA1c was 11.8% (IQR = 9-14), and only 10% had HbA1c < 7.5%. The analysis showed that there was a significant correlation between regular home blood glucose monitoring and glycemic control (*p* ≤ 0.001). However, there was no statistically significant correlation between glycemic control and insulin regimen (*p* = 0.73), as well as insulin dose (*p* = 0.74).

Regarding insulin treatment, most of the participants (95%) were on a conventional insulin regimen, 5% were on an intensive regimen, none of the patients were using an insulin pump. The median (IQR) insulin dose in unit/kg was 0.9 (IQR = 0.8-1.1).

### 3.2. Microvascular Complications

The presence of incipient diabetic nephropathy (persistent microalbuminuria) was observed in 36% of the participants. The youngest patient with microalbuminuria aged 11 years. Two patients had persistent macroalbuminuria (albuminuria more than 200 mg/l). Diabetic retinopathy was observed in 33% of the patients. The youngest patient with diabetic retinopathy aged 10 years and 7 months. Most of the patients with diabetic retinopathy had very mild nonproliferative diabetic retinopathy (66.7%). 30% had mild nonproliferative diabetic retinopathy. Only one patient (0.3%) had moderate nonproliferative retinopathy. Eleven percent of the patients were found to have both diabetic nephropathy and retinopathy. The youngest of them was 11 years old.

Out of the total study participants, only six patients were prepubertal. All of them had normal blood pressure. However, one of them had both microalbuminuria and bilateral very mild diabetic retinopathy. This patient whose age was 11 years had diabetes duration of only 3 years. Another patient whose age was 10 and a half with diabetes for 2 years had unilateral very mild diabetic retinopathy. This last patient had hypothyroidism, was overweight, and had a family history of T1DM and essential hypertension.

Characteristics of patients with and without any of the two microangiopathies are described in Table 1. As shown, patients with diabetic retinopathy had significantly longer duration of diabetes (*p* = 0.02) and higher HbA1c (*p* = 0.009) compared to patients without retinopathy. However, these two factors were not significantly different between groups with and without DN. There was no statistically significant difference between patients with and without any of the two microangiopathies regarding age, ethnic group, age at diabetes onset, BMI, puberty, and blood pressure.

### 3.3. Logistic Regression Analysis of Risk Factors

In a logistic regression analysis, considering the presence of DN to be the dependent variable, blood pressure (OR = 6.89, 95%CI = 1.17-40.52, *p* = 0.03) was a predictor for the presence of DN, while the only variable that predicts DR was HbA1c level (OR = 0.83, 95%CI = 0.68-1.00, *p* = 0.04). Age, duration, ethnic group, BMI, and the presence of the other microangiopathy were not associated with any of the complications (Table 2).

## 4. Discussion

This study was the first of its type, to the authors' knowledge, to be carried out in Sudan examining the frequency of diabetic nephropathy and retinopathy and their associated risk factors in this age group. As estimated, the incidence of T1DM in Sudan is rising from 9.5/100,000 in 1991 [26] to 10.1/100,000 in 2015 [27]. Diabetes has a profound impact on children and families, both emotionally and economically, particularly in the developing world. Poor control leads to future microvascular complications. Most mortality in a cohort of South African patients with T1DM was as a result of diabetic nephropathy [28].

This cross-sectional study involved both rural and urban populations who belong to the three main Sudanese ethnic groups: Nilo-Saharan, Afro-Asiatic, and Niger-Congo [19]. Most of the participants in the present study were Afro-Asiatic (79%), so the three ethnic groups were not equally represented in this study.

Regarding metabolic control, most of the participants (90%) were not achieving the target control (HbA1c less than 7.5%) [23]. This finding is common in African studies that involved old children and adolescents with T1DM [15, 16, 29, 30]. However, diabetes control is universally found to be worse in adolescent patients compared to younger children [29].

In the current study, the poor glycemic control as measured by HbA1c was statistically strongly related to the lack of regular home blood glucose monitoring, caused by the unavailability of sustained glucometer's strip supply (*p* ≤ 0.001). Although most of the patients are supplied with glucometers for free by the Sudan Childhood Diabetes Association (95% of the study group had a glucometer), most of the families could not afford to purchase the test strips as prices have increased tremendously over the last few years. In African cohort studies on children with diabetes, better metabolic control was achieved by frequent self-monitoring of blood glucose [17, 31]. However, poor glycemic control in the current study was associated with neither the insulin dose per kilogram nor the insulin regimen. This might be explained by the homogeneity of our participants regarding the insulin regimen since the vast majority (95%) were on conventional insulin. All types of insulin are provided for free funded by the Sudan Childhood Diabetes Association except insulin glargine. As insulin analogs are expensive and there is limited supply of NPH, most patients are put on premixed insulin alone or plus short-acting human insulin. This explains why most of our patients are on a conventional regimen. The trend in the last decade toward better diabetes control has been associated with the availability of frequent self-monitoring of blood glucose (SMBG), the use of insulin analogs, and more intensive management of diabetes including insulin pumps [29], things that are hard to afford in developing economics. The situation in this study is not different from that in other sub-Saharan countries where neither analog insulin nor multiple daily test strips are currently affordable [16, 29, 30, 32, 33].

One of the most noteworthy findings of this study is the high prevalence of microvascular chronic complications at a young age. Diabetic nephropathy was present in 36%, and diabetic retinopathy in 33% of the patients. Altogether, 22% had isolated diabetic retinopathy, 25% had isolated diabetic nephropathy, and 11% had both diabetic retinopathy and nephropathy. Thus, almost half of the patients (58 patients, 58%) had either one or both of the complications, and only 42 (42%) had neither of these complications. These findings concur with other African reports [15–17]. Among studies done in sub-Saharan Africa for instance, the observed prevalence of incipient diabetic nephropathy in this study is close to that in a Tanzanian study with comparable sample size and diabetes duration [16]. The DN frequency in this study is also comparable to the results of a recent Nigerian study carried out on a smaller number of patients with T1DM in the same age range as in this study [15]. Regarding the prevalence of DR in the current study (33%), it is slightly higher than the prevalence obtained by these two above-mentioned studies, which reported prevalence rates of 22.7% [16] and 16.7% [15] in their participants, respectively. This could be attributed to the inclusion of younger patients with shorter duration in the Tanzanian study [16] or to the use of fundoscopy to diagnose DR in both of the studies. It is known that fundoscopy is less sensitive than fundus photography in detecting the early stages of DR [34]. Therefore, securing fundus cameras if possible is recommended to detect more cases of early retinopathy as what happened in our study. Provision of fundus cameras for the screening of a common preventable cause of blindness like DR is cost-effective, especially in a poor country like Sudan. DN prevalence in the current study is also not far from that obtained by a Rwandan study [17].

The prevalence of microalbuminuria in the current study is much higher than that in a study carried out in Egypt which included wide age range participants with better glycemic control than ours [35]. These two factors in plus the use of fundoscopy could also explain the lower DR prevalence than that of our study.

The frequency of both of the microvascular complications in our study is higher than those reported from the developed world mostly due to better diabetes control and possibly genetic or ethnic variation [1, 9, 34, 36–38].

In line with previous similar studies [15, 16, 35], almost all the patients with DR in this study were at the stage of mild or very mild nonproliferative diabetic retinopathy, except one patient who had moderate nonproliferative DR.

In this study, 11 (11%) had combined diabetic retinopathy and nephropathy. No statistically significant relationship between the presence of diabetic nephropathy and retinopathy was found, a finding that contradicts many studies [9, 13, 39]. To explain this, further studies are needed as it may be due to different risk factors for these two microvascular complications in our population.

Out of the total study participants, seven percent of patients had hypertension and 8% had prehypertension, a prevalence that is not very different from the prevalence of hypertension in healthy Sudanese school children [40]. Compared to the previously reported prevalence of hypertension among adolescents with T1DM, it is not far from those in some previous studies [1, 41], but higher than others [42, 43] and lower than that obtained by one study [37]. This variation in the prevalence of hypertension among young patients with T1DM might be explained by other confounding factors like difference in the prevalence of obesity, lifestyle, genetics, or another unknown factor. In this study, 57% of the patients with hypertension and 50% of the patients with prehypertension had microalbuminuria. There was no statistically significant difference regarding the blood pressure status between the groups with and without DN. However, when logistic regression was done, high blood pressure was found to be a predictor of microalbuminuria (*p* = 0.03). High blood pressure association with DN was found by many studies [1, 8, 10, 13, 37, 38, 42]. However, it is not yet known which parameter of the blood pressure heralds the occurrence of microalbuminuria. Perhaps, it is the elevation from the baseline blood pressure, not the high blood pressure itself [44, 45]. Therefore, it is important to check and follow the blood pressure of young patients with T1DM to pick up the early elevation of blood pressure. In places with limited resources like Sudan, this can be an easy and early predictor of diabetic nephropathy. Since this is a cross-sectional study, it is hard to know whether high blood pressure had developed concomitantly, after or before microalbuminuria. Whatever the case, these groups of patients need close control of their blood pressure to halt the progress of nephropathy, as it is well known that monitoring and early treatment of hypertension prevent the progression of nephropathy [6, 46]. No significant relationship between high blood pressure and DR was found in this study, a finding that is in concordance with a previous study [9], but in contradistinction to others [8, 12, 13, 42, 47]. The role of BP in the development of early retinopathy in adolescents with T1DM was studied by Gallego et al. who concluded that both systolic and diastolic blood pressure are predictors of DR independently of incipient nephropathy [12]. However, there may be different pathogenic mechanisms for DR in our population.

No relationship between the presence of any of the two microangiopathies and ethnicity was found in this study; this might be attributed to the fact that the participants were homogeneous regarding ethnic groups, as the majority (79%) belong to the Afro-Asiatic group. The role of ethnicity and genetics in the development of diabetic microvascular complications had been suggested by many studies that showed geographic as well as ethnic variation in the susceptibility to these complications [5, 6, 14, 48]. Other studies suggested that these observed differences may be linked primarily to the unfavourable social and economic conditions that worsen the risk of poor blood glucose and blood pressure control [49, 50]. Another study attributed this difference to a mixture of genetic and socioeconomic factors that delay access to care [4]. Nevertheless, the role of genetics in susceptibility to nephropathy, retinopathy, and other diabetic complications largely remains to be explored.

Moreover, no relation between age at onset and DN or DR was found, a finding that is in agreement with some studies [6, 9], but in contradiction to other studies [8–10, 38]. This could be due to the interaction with other factors like length of prepubertal duration, age at puberty, or other unknown factors.

This study was relatively small and cross-sectional in design, yet it was able to show that patients with DR had significantly longer duration than patients without DR. However, the duration was not a predictor for DR when logistic regression was made. Many studies concluded that diabetes duration is a risk factor for DR development [10, 15, 47]. Moreover, some studies found that long diabetes duration is the major risk factor for the development of diabetic retinopathy in T1DM [9, 13, 36, 42, 47]. An association between duration of diabetes and diabetic nephropathy was not found in this study in keeping with other studies [6, 15, 16, 39, 51, 52]. However, contradictory results were found by others [1, 9, 38, 42, 53]. In fact, one study concluded that given sufficient diabetes duration, retinopathy is almost universal, but not all patients develop nephropathy [54].

Lastly, regarding glycemic control, patients with DR had significantly higher HbA1c than those without DR, and high HbA1c is a predictor for DR in the logistic regression, an observation that was also demonstrated by the landmark DCCT/EDIC [55] and numerous previous studies [5, 8, 9, 15, 42, 56]. Lack of appropriate glycemic control especially in the peripubertal years was found to be a significant risk factor for the onset and progression of DR by one recent African study [15].

This study did not demonstrate any significant difference in glycemic control between groups with and without nephropathy so do most other available studies from sub-Saharan Africa [4, 6, 15, 44, 57]. In a longitudinal study done in the Rwandan youth, the microalbuminuria rate did not significantly change even after a significant improvement in glycemic control [17]. This might be explained by the hypothesis that other factors such as high blood pressure or genetic predisposition may contribute more to the development of diabetic nephropathy [9]. An additional explanation could be that the relationship between hyperglycemia and the development of DN is not straightforward. Some studies found that it is the earlier poor glycemic control during the first few years of the disease course that is associated with the development of microalbuminuria [9]. Other studies hypothesized that hyperglycemia is a necessary but not sufficient factor for the development of diabetic nephropathy [39]. Nevertheless, the relationship between glycemic control and the development of DN in our population has to be fully elucidated by further studies.

It is known that the risk factors involved in the development and progression of different diabetic microangiopathies in T1DM are overlapping but not identical [58]. Additionally, it is hypothesized that genetic factors modulate the effects of different risk factors [5]. This might explain why some risk factors were found to be significant for one microangiopathy but not for the other. However, the root cause of this discrepancy needs further large prospective studies.

In conclusion, in the present study, a high prevalence of early signs of diabetic nephropathy and retinopathy was demonstrated among children and adolescents at a relatively young age and short duration of T1DM, since more than half of the individuals included in this study suffered from at least one of these two chronic complications attributable to diabetes. The fact that the majority of our participants had poor glycemic control could partially explain this high prevalence. High blood pressure was found to be a predictor for diabetic nephropathy. Associations of diabetic retinopathy with longer disease duration and poor glycemic control were verified. However, no such association was found for diabetic nephropathy.

This is the first exploratory study of diabetes-related microvascular complications in Sudanese children and adolescents. The rates we had found are high and suggest that health policymakers should prioritize diabetes care as a major component of health provision in Sudan to minimize the development of such costly and life-threatening complications, since the early stages of microvascular complications such as nephropathy and retinopathy are often asymptomatic. The primary clinical care emphasis on the prevention of vision loss or renal impairment must be appropriately directed at good glycemic control, early identification, accurate classification, and timely treatment of these complications. In this study, the proportion of patients with poor glycemic control was high, and therefore, focusing on glycemic control would be of great benefit. Large prospective studies are also necessary to validate the current findings.

## Figures and Tables

**Table 1:** Characteristics of the total participants and patients with and without nephropathy and retinopathy.

Risk factor	Total subjects	No DN	DN	*p* value	No DR	DR	*p* value
*N* (%)	100 (100%)	64 (64%)	36 (36%)	—	67 (67%)	33 (33%)	—
Median age (years)	15.6 (14.1-17)	15.4 (13.9-16.9)	15.9 (14.4-17.2)	0.33	15.3 (14-16.9)	15.7 (14.7-17.3)	0.33
Male/female (%)	39/61 (39%/61%)	22/42 (34.4%/65.6%)	17/19 (47.2%/52.8%)	0.21	26/41 (38.8%/61.2%)	13/20 (39.4%/60.6%)	0.95
Residence: urban/rural	57/43 (57%/43%)	38/26 (59.4%/40.6%)	19/17 (52.8%/47.2%)	0.52	36/31 (53.7%/46.3%)	21/12 (63.6%/36.4%)	0.35
Ethnic group							
Afro-Asiatic	79 (79%)	48/64 (75%)	31/36 (86.1%)	0.40	50/67 (74.7%)	29/33 (87.8%)	0.3
Nilo-Saharan	12 (12%)	9/64 (14%)	3/36 (8.3%)		10/67 (14.9%)	2/33 (6.1%)	
Niger-Congo	9 (9%)	7/64 (11%)	2/36 (5.6%)		7/67 (10.4%)	2/33 (6.1%)	
Median age at diabetes diagnosis (years)	9 (7-11.4)	9.1 (5.5-11.5)	9 (7-11.3)	0.94	9 (7-12)	8.7 (5.3-11)	0.15
Median diabetes duration (years)	6 (4-9)	6 (4-8.7)	6.6 (4-9)	0.76	5.3 (4-8)	7 (5.1-10.2)	0.02
BMI (overweight+obese/total)	9/100 (9%)	6/64 (9.4%)	3/36 (8.3%)	0.43	6/67 (9%)	3/33 (9%)	0.45
Puberty (pubertal/total)	94/100 (94%)	59/64 (92.2%)	35/36 (97.2%)	0.18	63/67 (94%)	31/33 (94%)	0.47
Blood pressure							
Normal	85/100 (85%)	58/64 (90.6%)	27/36 (75%)	0.07	57/67 (85%)	28/33 (85%)	0.76
Prehypertension	8/100 (8%)	4/64 (6.3%)	4/36 (11.1%)		6/67 (9%)	2/33 (6%)	
Hypertension	7/100 (7%)	2/64 (3.1%)	5/36 (13.9%)		4/67 (6%)	3/33 (9%)	
Median HbA1c (%)	11.8% (9-14)	11.7 (9-13.3)	12 (9-14)	0.75	10.2 (8.6-12)	12.4 (9.1-14)	0.009

**Table 2:** Results of logistic regression analysis for risk factors of microvascular complications.

Predictor	Diabetic nephropathy		Diabetic retinopathy	
	Odds ratio (95% CI)	*p* value	Odds ratio (95% CI)	*p* value
Age	1.16 (0.91-1.48)	0.23	1.14 (0.88-0.88)	1.49
Ethnic group	0.40 (0.08-1.99)	0.26	0.57 (0.10-3.16)	0.52
Diabetes duration	1.02 (0.88-1.18)	0.81	1.12 (0.97-1.30)	0.13
BMI class	0.43 (0.13-1.43)	0.17	2.05 (0.57-7.41)	0.27
BP class	6.89 (1.17-40.52)	0.03	0.65 (0.10-4.36)	0.66
The most recent HbA1c	1.07 (0.89-1.29)	0.47	0.83 (0.68-1.00)	0.04
Presence of the other complication (DN/DR)	0.75 (0.28-2.05)	0.58	0.72 (0.26-2.01)	0.53

## Data Availability

The data of this research will be available on request.

## Abbreviations

BP: Blood pressure
CDC: Centers for Disease Control and Prevention
DCCT: Diabetes Control and Complications Trial
DM: Diabetes mellitus
DN: Diabetic nephropathy
DR: Diabetic retinopathy
EDIC: Epidemiology of Diabetes Interventions and Complications
IQR: Interquartile range
ISPAD: International Society for Pediatric and Adolescent Diabetes
NPDR: Nonproliferative diabetic retinopathy
T1DM: Type 1 diabetes mellitus.
